# Analysis of sodium phenylbutyrate and taurursodiol survival effect in ALS using external controls

**DOI:** 10.1002/acn3.51915

**Published:** 2023-10-09

**Authors:** Sabrina Paganoni, Melanie Quintana, Alexander V. Sherman, Matteo Vestrucci, Yuehui Wu, Jamie Timmons, Merit Cudkowicz, the Pooled Resource Open‐Access ALS Clinical Trials Consortium

**Affiliations:** ^1^ Sean M. Healey and AMG Center for ALS & the Neurological Clinical Research Institute, Massachusetts General Hospital, Harvard Medical School Boston Massachusetts USA; ^2^ Spaulding Rehabilitation Hospital, Harvard Medical School Boston Massachusetts USA; ^3^ Berry Consultants, LLC Austin Texas USA; ^4^ Amylyx Pharmaceuticals, Inc. Cambridge Massachusetts USA

## Abstract

**Objective:**

Sodium phenylbutyrate and taurursodiol (PB and TURSO) was evaluated in amyotrophic lateral sclerosis (ALS) in the CENTAUR trial encompassing randomized placebo‐controlled and open‐label extension phases. On intent‐to‐treat (ITT) survival analysis, median overall survival (OS) was 4.8 months longer and risk of death 36% lower in those originally randomized to an initial 6‐month double‐blind period of PB and TURSO versus placebo. To estimate PB and TURSO treatment effect without placebo‐to‐active crossover, we performed a post hoc survival analysis comparing PB and TURSO‐randomized participants from CENTAUR and a propensity score–matched, PB and TURSO‐naïve external control cohort from the Pooled Resource Open‐Access ALS Clinical Trials (PRO‐ACT) database.

**Methods:**

Clinical trial control participants from the PRO‐ACT database who met prespecified eligibility criteria were propensity score matched 1:1 with PB and TURSO‐randomized CENTAUR participants using prognostically significant covariates in ALS.

**Results:**

Baseline characteristics including propensity score–matched covariates were generally well balanced between CENTAUR PB and TURSO (*n* = 89) and PRO‐ACT external control (*n* = 85) groups. Estimated median (IQR) OS was 23.54 (14.56–39.32) months in the CENTAUR PB and TURSO group and 13.15 (9.83–19.20) months in the PRO‐ACT external control group; hazard of death was 52% lower in the former group (hazard ratio, 0.48; 95% CI, 0.31–0.72; *p* = 0.00048).

**Interpretation:**

This analysis suggests potentially greater survival benefit with PB and TURSO in ALS without placebo‐to‐active crossover than seen on ITT analysis in CENTAUR. Analyses using well‐matched external controls may provide additional context for evaluating survival effects in future ALS trials.

## Introduction

The efficacy and safety of an oral, fixed‐dose combination of sodium phenylbutyrate and taurursodiol (PB and TURSO [RELYVRIO^®^; Amylyx Pharmaceuticals, Inc., Cambridge, MA]) in amyotrophic lateral sclerosis (ALS) were evaluated in the CENTAUR trial encompassing a 6‐month randomized placebo‐controlled phase and an open‐label extension (OLE) long‐term follow‐up phase in which participants were treated with PB and TURSO. As expected over a 6‐month duration, the number of deaths was low in the randomized phase of CENTAUR and statistically similar between the PB and TURSO group and placebo group.^1^ In a long‐term intent‐to‐treat (ITT) survival analysis that spanned both the randomized and OLE phases (longest post‐randomization follow‐up, 42 months) and incorporated all randomized participants, median overall survival was 4.8 months longer and risk of death 36% lower (hazard ratio [HR], 0.64; 95% CI, 0.42–1.00; *p* = 0.048) in those originally randomized to PB and TURSO versus placebo. Notably, 71% of participants originally randomized to placebo enrolled in the OLE phase and thus crossed over to active treatment after 6 months.^2^

OLE phases provide an opportunity for longer‐term assessment of survival outcomes in trials with randomized placebo‐controlled phases of short duration in addition to increasing access to investigational therapies for people living with fatal conditions such as ALS. However, placebo‐to‐active crossover in trials incorporating OLE phases may lead to underestimation of the effect of these therapies on overall survival in ITT analyses.^3^, ^4^ External controls provide a means for estimating treatment effect in the absence of a treatment‐naïve randomized control group. However, to reduce bias relating to the use of external controls, careful consideration should be given to their source as well as statistical methods that impose balance between the external control and active comparator groups.^5^

The Pooled Resource Open‐Access ALS Clinical Trials (PRO‐ACT) database is a robust resource of open‐access data relating to clinical outcomes in ALS,^6^ offering a potential source for external controls in analyses of ALS clinical trial data. PRO‐ACT is the largest ALS clinical trials database in existence, incorporating anonymized longitudinal data from 11,675 participants in 29 Phase 2 and 3 ALS clinical trials.^6^, ^7^ To estimate the treatment effect of PB and TURSO on survival in the absence of placebo‐to‐active crossover, we performed a post hoc survival analysis comparing PB and TURSO‐randomized participants from CENTAUR (hereafter referred to as ‘CENTAUR PB and TURSO group’) and a matched external PB and TURSO‐naïve cohort from the PRO‐ACT database (hereafter referred to as ‘PRO‐ACT external control group’).

## Methods

### Analysis cohorts

During the conduct of the CENTAUR trial, eligible participants were in the randomized phase from June 2017 to September 2019 and in the OLE phase from March 2018 to March 2021. CENTAUR trial eligibility criteria allowed for the stable use of riluzole and edaravone before and during the trial. Survival results for the CENTAUR PB and TURSO group in this analysis were obtained from the aforementioned ITT analysis, the methods of which have been previously described.^2^, ^8^ Vital status was ascertained as of March 2021 for all but one participant in the CENTAUR PB and TURSO group, who was censored at the date of last contact.^2^

**Figure 1:**
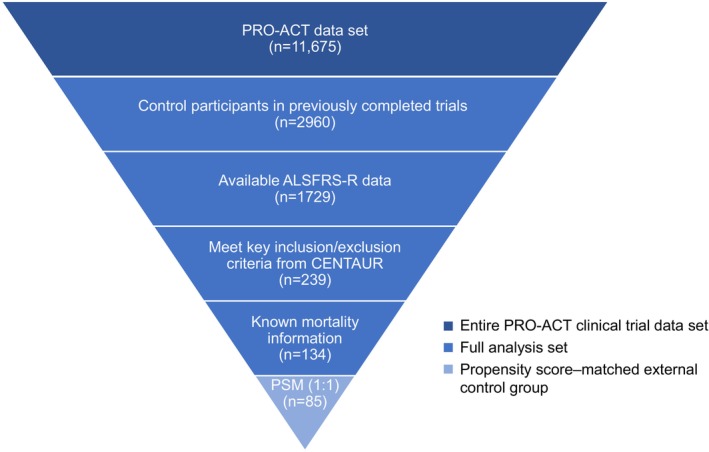
Summarized process for constructing the PRO‐ACT external control group. ALSFRS‐R, Amyotrophic Lateral Sclerosis Functional Rating Scale‐Revised; PRO‐ACT, Pooled Resource Open‐Access ALS Clinical Trials; PSM, propensity score matching.

Data from the PRO‐ACT database that were used in the preparation of this article have been volunteered by PRO‐ACT Consortium members. The process for constructing the PRO‐ACT external control group is schematically summarized in Figure 1. Data for this analysis were downloaded from the PRO‐ACT website (www.alsdatabase.org) and comprise the latest version of the data set from August 2022. These clinical trials took place over a period of 25 years, from 1994 to 2019. The external control group was constructed from the full analysis set consisting of clinical trial participants from the PRO‐ACT database who were control participants in their respective trials, had baseline and at least one post‐baseline Amyotrophic Lateral Sclerosis Functional Rating Scale‐Revised (ALSFRS‐R) total scores recorded, had known mortality information, and met major eligibility criteria from CENTAUR, including age of 18–80 years, diagnosis of definite ALS based on revised El Escorial criteria,^9^ duration of ≤18 months since symptom onset, and a forced or slow vital capacity >60% of predicted value at screening.

To account for potential imbalance in baseline participant characteristics and other variables across trials,^10^, ^11^ the CENTAUR PB and TURSO group and the PRO‐ACT external control group were propensity score matched adjusting for baseline covariates of known prognostic significance in ALS, including time since symptom onset^12^; pre‐baseline ALSFRS‐R slope, defined as the rate of change in ALSFRS‐R total score from symptom onset to study baseline^13^; and vital capacity^14^ and age^12^ at study baseline. Propensity score matching was conducted in a 1:1 ratio using a prespecified caliper width of 0.4 of the standard deviation of the logit of the propensity score.^15^

### Standard protocol approvals, registrations, and participant consents

The clinical study protocol for CENTAUR was approved by a central institutional review board (the Partners Human Research Committee) for all trial sites, and participants provided written informed consent before entering each trial phase.^1^ The CENTAUR trial was registered with ClinicalTrials.gov (randomized phase: NCT03127514; OLE phase: NCT03488524). For all trials that generated the data included in PRO‐ACT, trial protocols were approved by the participating medical centers, and all participants gave informed consent. Data from these trials were provided to PRO‐ACT for research purposes only and under explicit conditions that all users of the data would maintain participants' anonymity. In cases where donated data were not already anonymized when submitted to PRO‐ACT, the data were anonymized by the bioinformatics team at the Neurological Clinical Research Institute (NCRI) of the Massachusetts General Hospital following the Health Insurance Portability and Accountability Act anonymization conventions prior to becoming public.^7^ All data in the PRO‐ACT database are de‐identified; therefore, it was not possible to locate participants and obtain consent again for this analysis.

### Statistical analysis methods

Overall survival analysis was conducted for 1:1 propensity score–matched individuals from the PRO‐ACT external control group and the CENTAUR PB and TURSO group. Overall survival (time to death) was defined as the time from date of randomization to date of death due to any cause. Median overall survival and IQRs were estimated for the two cohorts using the Kaplan–Meier method. The HR between the two groups was estimated from a Cox proportional hazards model. The treatment difference in overall survival between the two groups was declared significant if the two‐sided *p* value for test on HR of the Cox proportional hazards model was ≤0.05.

Sensitivity analyses were performed with different caliper widths for the propensity score matching (0.1, 0.2, and 0.6, in addition to the prespecified caliper width of 0.4), on the full eligible PRO‐ACT analysis set without propensity score matching, and on the full eligible PRO‐ACT analysis set with propensity score inverse probability treatment weighting (IPTW). In addition, change in ALSFRS‐R total score from baseline through 24 weeks was assessed in the propensity score–matched PRO‐ACT external control group with an aim of evaluating the comparability of change in this functional end point to that in the placebo group from the randomized phase of CENTAUR.

## Results

A total of 134 individuals in PRO‐ACT met inclusion criteria for this analysis and had known mortality information (full analysis set; Fig. 1). Of 89 participants in the CENTAUR PB and TURSO group, 85 had a match in the PRO‐ACT external control group on 1:1 propensity score matching using a caliper width of 0.4. Baseline demographic and clinical characteristics including covariates used for propensity score matching were generally well balanced between groups (Table 1). Functional outcome, as measured by the mean change in ALSFRS‐R total score from baseline through 24 weeks, was comparable between the PRO‐ACT external control group (−1.66 points/month) and the placebo group (−1.66 points/month) in the randomized phase of CENTAUR (Table 2).^1^

**Table 1:** Baseline characteristics.

Characteristic	CENTAUR PB and TURSO group (*n* = 89)	PRO‐ACT external control group (*n* = 85)
Age^a^ ^,^ ^b^, years	57.9 (10.6), 31.0–79.0	57.2 (9.5), 33.8–75.6
Bulbar onset, *n* (%)	26 (29.2)	22 (25.9)
Riluzole use, *n* (%)	61 (68.5)	58 (68.2)
Pre‐baseline ALSFRS‐R slope^a^, points/month	0.96 (0.42), 0.12–1.94	0.91 (0.53), 0.12–3.03
VC^a^ ^,^ ^b^, percent predicted value	83 (19), 38^c^–142	84 (13), 60–131
Baseline ALSFRS‐R total score	35.6 (5.73), 18.0–46.0	36.8 (5.35), 26.0–47.0
Time since ALS symptom onset^a^, months	13.6 (3.8), 3.0–20.0	13.0 (3.4), 4.1–17.6
Time since ALS diagnosis, months	5.9 (3.32), 1.3–15.7	5.2 (3.04), 0.5–14.1

Data are presented as mean (SD), range unless otherwise noted.

ALS, amyotrophic lateral sclerosis; ALSFRS‐R, Amyotrophic Lateral Sclerosis Functional Rating Scale‐Revised; PB and TURSO, sodium phenylbutyrate and taurursodiol; PRO‐ACT, Pooled Resource Open‐Access ALS Clinical Trials; SD, standard deviation; VC, vital capacity (forced or slow).

^a^ Denotes covariate used for propensity score matching.

^b^ At study baseline.

^c^ This represents the minimum value at baseline. All participants met VC criteria for trial inclusion at screening.

**Table 2:** Change in ALSFRS‐R total score progression rate from baseline through 24 weeks: CENTAUR trial population versus PRO‐ACT external control group.

	Originally randomized treatment group in CENTAUR^1^		PRO‐ACT external control group (*n* = 85)
	PB and TURSO (*n* = 87)^a^	Placebo (*n* = 48)	
Mean (SE) ALSFRS‐R progression rate, points/month	−1.24 (0.120)	−1.66 (0.159)	−1.66 (0.137)

ALSFRS‐R, Amyotrophic Lateral Sclerosis Functional Rating Scale‐Revised; ITT, intent‐to‐treat; PB and TURSO, sodium phenylbutyrate and taurursodiol; PRO‐ACT, Pooled Resource Open‐Access ALS Clinical Trials; SE, standard error.

^a^ Modified ITT population, which excluded two participants who died soon after randomization.

**Figure 2:**
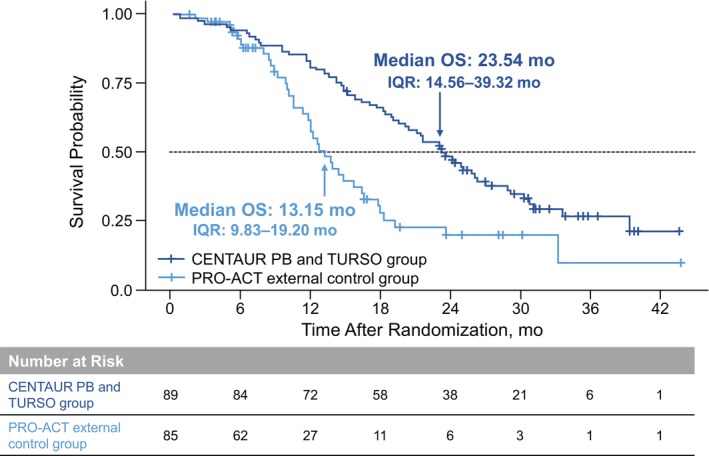
Kaplan–Meier analyses: CENTAUR PB and TURSO group and PRO‐ACT external control group. Kaplan–Meier plot for the CENTAUR PB and TURSO group is from the ITT analysis previously conducted at a cutoff date coinciding with the final participant visit in CENTAUR.^2^ ITT, intent‐to‐treat; OS, overall survival; PB and TURSO, sodium phenylbutyrate and taurursodiol; PRO‐ACT, Pooled Resource Open‐Access ALS Clinical Trials database.

Compared with the observed median (IQR) overall survival of 23.54 (14.56–39.32) months in the CENTAUR PB and TURSO group,^2^ median (IQR) overall survival through 42 months of follow‐up was 13.15 (9.83–19.20) months in the PRO‐ACT external control group (difference, 10.39 months). Hazard of death was 52% lower in the CENTAUR PB and TURSO group^2^ versus the PRO‐ACT external control group (HR, 0.48; 95% CI, 0.31–0.72; *p* = 0.00048; Fig. 2). Sensitivity analyses using different caliper widths, on the full‐eligible PRO‐ACT analysis set without propensity score matching, and on the full eligible PRO‐ACT analysis set with propensity score IPTW showed consistent results with the main analysis (Table S1).

## Discussion

To estimate the treatment effect of PB and TURSO on survival in ALS in the absence of the placebo‐to‐active crossover that occurred in the OLE phase of the CENTAUR trial, we compared overall survival in those randomized to PB and TURSO with an external, PB and TURSO‐naïve control group from PRO‐ACT, the largest ALS clinical trials data set. Median overall survival was 10.39 months longer in those originally randomized to PB and TURSO in CENTAUR versus the external control group in this analysis, whereas the ITT analysis of the CENTAUR trial showed that median overall survival for PB and TURSO was 4.8 months longer versus placebo. The results of the current analysis align with a previous analysis using a rank‐preserving structural failure time model (RPSFTM), an advanced statistical method that adjusts overall survival for the effect of treatment crossover in the placebo group within a clinical trial incorporating a crossover design.^3^, ^4^ In the RPSFTM analysis of CENTAUR, median overall survival was 9.7 months longer in the group originally randomized to PB and TURSO compared with RPSFTM‐adjusted placebo.^2^

Though less preferable than a concurrent, randomized control group given the potential for introducing bias, utilization of control data from an external database provides a treatment‐naïve comparator option for active treatment in clinical trials that allow for placebo‐to‐active crossover or completely exclude a concurrent placebo group based on ethical concerns.^5^, ^16^ By reducing the number of participants required for enrollment, use of external control data may also hasten drug development^16^ and has even been applied in a limited capacity within pivotal trials leading to regulatory approvals, most often for therapies for rare diseases.^5^, ^17^

To minimize bias due to imbalance among nonrandomized cohorts or other trial variables, external controls should be as closely matched to the investigational cohort as possible.^5^ In the case of our analysis, the PRO‐ACT database was a relevant source for external controls, given that many of the trials represented in PRO‐ACT were conducted at sites overlapping with those represented in CENTAUR. In addition, in line with seminal recommendations pertaining to use of external controls,^18^ the controls in our analysis were selected based on similar eligibility criteria as used for the investigational cohort and on well‐known prognostic factors in ALS,^5^ with the latter serving as the basis for propensity score matching between the CENTAUR and external cohorts.

Propensity score matching has been proposed by the US Food and Drug Administration as a means of improving the quality of external control data^10^ and has been used in analyses supporting regulatory approval of drugs for rare conditions that employed external controls.^17^ In observational studies in cardiology, propensity score matching was shown to be superior to other methods that are used to adjust for confounding, particularly when covariate imbalance was not prominent.^19^, ^20^ Indeed, the accuracy of propensity score matching depends greatly on the prognostic variables used for matching and the similarity between the external control group and study group.^11^ Utilization of propensity score matching was deemed appropriate in the setting of our analysis, given that variables predicting survival in ALS are well known and, as previously mentioned, PRO‐ACT provided a very similar study setting compared to CENTAUR. Matches were found for 85 of the 89 participants in the CENTAUR PB and TURSO group using the selected caliper width of 0.4. Results of the survival analysis were similar regardless of caliper width. The results of the main analysis were further supported by sensitivity analyses performed without propensity score matching and with propensity score IPTW. Finally, the comparability of the PRO‐ACT external control group and the overall CENTAUR population was supported by examination of the change in ALSFRS‐R total score progression rate over similar periods in the PRO‐ACT external control group and in the placebo group in the randomized phase of CENTAUR; however, these findings must be interpreted with caution as these groups were not systematically compared in a clinical trial setting.

Because data from clinical trials are generated in a controlled setting, the use of the PRO‐ACT data set in our analysis provided high‐quality external control data, in contrast to external data obtained from differing sources with varying data quality. Our analysis was also strengthened by the focus on survival, an objective end point that could be consistently and precisely measured in both analysis groups.^5^ However, death events may be missed in clinical trial participants because of loss to follow‐up. Hence, the eligibility criteria for the external control group in this analysis included the need for known mortality information to match the nearly complete ascertainment of survival in participants in CENTAUR, all but one of whom had confirmed vital status as of the analysis cutoff date. The requirement for known mortality information may have introduced bias toward shorter overall survival in the external control group, presuming confirmation of death is easier to ascertain at a given date than confirmation of being alive.

Our analysis also did not account for potential between‐group differences in the frequency of initiating respiratory support or gastrostomy tube placement, both potentially life‐extending interventions in ALS, as this information is not comprehensively captured in the PRO‐ACT database. Similarly, neurofilament levels are not included in the PRO‐ACT database and could not be used as a covariate for propensity score matching in our analysis. However, the groups were matched based on other baseline covariates that have been previously identified as significant predictors of survival duration in ALS, including time since symptom onset, pre‐baseline ALSFRS‐R, and age and vital capacity at baseline. In addition, the matched cohorts included similar proportions of participants with bulbar‐onset ALS and riluzole use, additional factors that have been shown to predict survival duration.^21^, ^22^ Finally, variations in clinical practice over time may have contributed to confounding of our analysis as well, given the wide‐ranging period for enrollment of clinical trial participants in the PRO‐ACT external control group. That said, median overall survival has remained in the range of 2.2–2.4 years after diagnosis in population‐based studies that assessed survival in people living with ALS who presented for care between 1998 and 2011.^23^, ^24^

In conclusion, the results of this analysis align with prior analyses using statistical models adjusting for placebo‐to‐active crossover in CENTAUR and suggest a potentially greater survival benefit with PB and TURSO in ALS than seen on ITT analysis. The ongoing global Phase 3 PHOENIX trial (ClinicalTrials.gov identifier NCT05021536) will evaluate the effect of PB and TURSO on a variety of outcomes, including survival, in a larger population of people living with ALS and for a longer duration than in CENTAUR. Analyses using external controls may provide additional context for survival outcomes in future trials in ALS. Adherence to robust methodology, particularly selection of optimally matched external controls, is pivotal when performing such analyses.

## Funding information

Funding for the CENTAUR trial was provided by Amylyx Pharmaceuticals, Inc., ALS Finding a Cure^®^, and The ALS Association. This analysis was funded by Amylyx Pharmaceuticals, Inc.

## Author Contributions

SP, MQ, AVS, MV, YW, JT, and MC contributed to the drafting and revision of the manuscript for content. All authors provided final approval of the manuscript for submission. SP, AVS, MC, and the PRO‐ACT Consortium played major roles in the acquisition of data. SP, MQ, AVS, MV, YW, JT, and MC analyzed and/or interpreted the data. MQ, MV, and YW conducted all statistical analyses.

## Conflict of Interest

S. Paganoni reports research grants from the National Institutes of Health, Alector Therapeutics, Biohaven, Cytokinetics, Anelixis Pharmaceuticals, Revalesio Corporation, UCB, Clene, Prilenia, Seelos Therapeutics, Calico, and Denali Therapeutics unrelated to this manuscript; consulting fees from Amylyx Pharmaceuticals, Frequency Therapeutics, SOLA Pharmaceuticals, Stealth BioTherapeutics, Orion, Roche, Janssen, and Arrowhead; honoraria from Medscape; and board membership in the Association of Academic Physiatrists. M. Quintana and M. Vestrucci are employees of Berry Consultants, LLC, report consulting fees from Amylyx Pharmaceuticals for some of the analyses described in the submitted work, and serve as consultants to numerous additional pharmaceutical and device companies. A.V. Sherman reports grants from the National Institutes of Health, US Food and Drug Administration, the ALS Association, and ALS Finding a Cure^®^ and has research contracts with Biogen, Amylyx, and Mitsubishi Tanabe Pharma America (MTPA). Y. Wu and J. Timmons are full‐time employees of and have stock options in Amylyx Pharmaceuticals, Inc. M. Cudkowicz reports consulting fees from Immunity Pharm Ltd, Cytokinetics, Takeda, Biogen, ALSpharma, RRD International, Transposon, QurAlis, Regeneron Pharmaceuticals, AB Science, Locust Walk, Servier, Vector Y, Roche, Novartis, Arrowhead Pharmaceuticals, VectorY Therapeutics, Servier, Pasithea Therapeutics, and Denali Therapeutics and serves on the board of directors for Praxis.

## Supporting information


Table S1
