# Supplementary material for: Analysis of sodium phenylbutyrate and taurursodiol survival effect in ALS using external controls
Source: Ann Clin Transl Neurol. 2023 Oct 9;10(12):2297–304. doi: 10.1002/acn3.51915 (PMC10723227; doi:10.1002/acn3.51915)
Supplement: Supplementary file 1 — Table S1 [file ACN3-10-2297-s001.docx]

**Table S1.** Sensitivity analyses using different caliper widths and on the full eligible PRO-ACT analysis set without propensity score matching and with propensity score IPTW.

| Analysis | PRO-ACT Matched Set, n | Median (IQR) OS, mo | | Median OS Difference, mo | HR, Mean  (95% CI)^a^ | 2-Sided *P* Value |
| --- | --- | --- | --- | --- | --- | --- |
|  |  | PB and TURSO Group  (n=89) | PRO-ACT Matched Set |  |  |  |
| Main analysis^b^ | 85 | 23.54  (14.56-39.32) | 13.15  (9.83-19.2) | 10.39 | 0.48  (0.31-0.72) | .00048 |
| Caliper 0.1 | 75 | 23.54  (14.56-39.32) | 13.15  (10.03-19.2) | 10.39 | 0.48  (0.31-0.74) | .00089 |
| Caliper 0.2 | 80 | 23.54  (14.56-39.32) | 13.15  (9.83-19.2) | 10.39 | 0.49  (0.32-0.76) | .00114 |
| Caliper 0.6 | 89 | 23.54  (14.56-39.32) | 13.15  (9.83-19.2) | 10.39 | 0.47  (0.31-0.71) | .00035 |
| Full analysis set | 134 | 23.54  (14.56-39.32) | 12.53  (9.93-18.21) | 11.01 | 0.46  (0.32-0.66) | .00003 |
| Full analysis set IPTW | 134 | 23.54  (14.56-39.32) | 12.56  (9.93-18.21) | 10.68 | 0.47  (0.32-0.7) | .00018 |

^a^From a Cox proportional hazards model.

^b^Propensity score matching using caliper width of 0.4 of the standard deviation of the logit of the propensity score.

HR, hazard ratio; IPTW, inverse probability treatment weighting; OS, overall survival; IQR, interquartile range; PB and TURSO, sodium phenylbutyrate and taurursodiol; PRO-ACT, Pooled Resource Open-Access ALS Clinical Trials.
